# Understanding client engagement in digital mental health interventions: An investigation of the eTherapy Attitudes and Process Questionnaire

**DOI:** 10.1002/jclp.23342

**Published:** 2022-03-10

**Authors:** Bonnie Clough, Christina Yousif, Sasha Miles, Sophia Stillerova, Aarthi Ganapathy, Leanne Casey

**Affiliations:** ^1^ School of Applied Psychology Griffith University Mount Gravatt Queensland Australia; ^2^ Menzies Health Institute Queensland Gold Coast Queensland Australia

**Keywords:** adherence, e‐mental health, engagement, internet interventions, theory of planned behavior

## Abstract

**Aims:**

Digital mental health services may increase the accessibility and affordability of mental health treatments. However, client dropout a low use is often reported. The purpose of the current study was to investigate the structural validity of the e‐Therapy Attitudes and Process (eTAP) questionnaire, as a theoretically based (theory of planned behavior) tool for understanding ongoing client engagement intentions with digital mental health treatments. The possible role of eHealth literacy in predicting behavioral intentions to use digital mental health treatments was also examined.

**Methods:**

Participants were 244 Australian‐based adults aged between 18 and 56 years, who were currently using a digital mental health tool. Data were collected via online survey.

**Results:**

Confirmatory Factor Analysis was conducted, with good model fit obtained following two theoretically supported modifications. Moderated hierarchical regression supported the utility of the theory of planned behavior in predicting behavioral intentions, with attitudes emerging as a strong and consistent individual predictor. No support was found for the moderating role or individual significance of eHealth literacy.

**Conclusions:**

These findings support the clinical and research use of the eTAP as a theory‐based measure to understand client engagement in digital mental health interventions. The study also highlights the need for interventions to target attitudes to improve clients' ongoing engagement in digital mental health.

## 1 INTRODUCTION

1.1

Mental health disorders affect more than 1 billion people each year and account for approximately 7% of the global burden of disease and 19% of all years lived with disability (Rehm & Shield, 2019). Prevalence of mental health difficulties rose in 2020 due to the effects of the Covid‐19 pandemic (Xiong et al., 2020), with a global mental health crisis predicted (The Lancet Infectious Diseases, 2020). Despite the high prevalence and costs associated with mental health disorders, treatment coverage remains poor. Before the Covid‐19 pandemic, mental health treatment coverage failed to exceed 50% in any country (Fairburn & Patel, 2017). Yet despite increased mental health prevalence and burden in 2020, the World Health Organisation reported disruption of mental health services in 93% of countries (World Health Organisation, 2020). There is a growing need for innovative models of mental health service delivery to enable cost‐effective, easily disseminable, and remote treatment delivery.

Digital mental health approaches have the capacity to meet this need, but there are concerns about long‐term user engagement. Dose‐response effects have been established in the field (Manwaring et al., 2008), with low client engagement diminishing the value of digital mental health approaches. A greater understanding of the factors which may enhance user engagement with digital mental health technologies is needed. A key requirement for this understanding is the availability of psychometrically validated tools for clinicians and researchers to understand user engagement. A newly developed measure, the eTherapy Attitudes and Process (eTAP) Questionnaire, has demonstrated strong initial psychometrics for this purpose, although validation beyond the initial study has yet to be reported. The scale was designed to be used in monitoring factors related to ongoing engagement (rather than initial uptake) in digital mental health interventions. The purpose of the present investigation was to provide further validation of the eTAP, specifically with regard to confirmation of structural validity. A secondary aim was to investigate the possible role of eHealth literacy in the prediction of behavioral intentions.

## 2 DIGITAL TECHNOLOGIES FOR MENTAL HEALTH TREATMENT

Digital mental health services have the capacity to reach more people, at low cost, in a confidential format, to overcome barriers associated with accessing traditional face‐to‐face psychological treatment (Karyotaki et al., 2015; Lattie et al., 2019). Considerable support exists for the efficacy of digital mental health approaches. Meta‐analytic research into digital cognitive behavior therapy (CBT) interventions has demonstrated symptom reductions in adults and children with anxiety and depressive symptoms, social phobia, and panic disorder symptoms (Andrews et al., 2010; Firth, Torous, Nicholas, Carney, Pratap, et al., 2017; Firth, Torous, Nicholas, Carney, Rosenbaum, et al., 2017; Hollis et al., 2017). Within telehealth approaches, videoconference psychotherapy is considered equally effective as face‐to‐face therapy in reducing psychological symptoms (Norwood et al., 2018). That digital mental health approaches can be efficacious and beneficial is now widely established and accepted. However, despite clear benefits, the potential for digital mental health uptake is yet to be fully realized (Hollis et al., 2017).

Digital mental health approaches have typically been associated with high levels of initial user access and engagement (Fleming et al., 2018). Yet issues with user attrition have been commonly reported (Fleming et al., 2018; Linardon & Fuller‐Tyszkiewicz, 2020; Richards & Richardson, 2012). Fleming et al. (2018) argued that for digital interventions to have an effect, users need to receive a “beneficial dose.” That is, treatment completion or sustained use is required. In Fleming et al.'s (2018) meta‐analysis of digital mental health program use, treatment completers or ongoing users ranged from less than 1% to 28%. In a meta‐analysis of computer‐based treatments for depression. Richards and Richardson (2012) reported dropout rates of 74%. Similarly, a meta‐analysis of smartphone interventions in mental health found just 34% of participants completed the treatment package (Linardon & Fuller‐Tyszkiewicz, 2020). In comparison, face‐to‐face drop rates for depression treatment have been estimated to range from 0% to 50% (Cooper & Conklin, 2015; Wierzbicki & Pekarik, 1993).

A number of hypotheses exist as to why digital mental health programs are often not used to completion. Application usability (Lattie et al., 2019), access to technology (Christensen et al., 2009), and the effect of therapist interactions in digital platforms (Andersson & Cuijpers, 2009; Beatty & Binnion, 2016) have been proposed as barriers to users' full engagement online. Beyond the laboratory, Fleming et al. (2018) found reduced adherence in the community in comparison to rates under research trial conditions. For digital mental health approaches to reach their full potential in supporting population‐level service provision, a greater understanding of factors influencing user engagement is needed.

### 2.1 Understanding client engagement in digital mental health

Research investigating client factors influencing ongoing digital mental health engagement has mainly focused on ad hoc variables such as demographic or other static variables (Karyotaki et al., 2015; Torous et al., 2018). Being male (Beatty & Binnion, 2016; Karyotaki et al., 2015) of a lower educational background (Karyotaki et al., 2015), and commencing a program with higher self‐reported mental health severity and/or diagnosis comorbidity (Al‐Asadi et al., 2014; Arnold et al., 2019; Karyotaki et al., 2015) significantly increases a user's risk of dropping out from digital mental health interventions. Despite a common assumption that younger age cohorts will better engage with digital mental health formats, research continues to show mixed results regarding the effect of age on digital mental health treatment adherence (Al‐Asadi et al., 2014; Arnold et al., 2019; Beatty & Binnion, 2016). While providing valuable insights into the users of digital mental health platforms, static variables only provide one perspective on understanding user engagement. Greater focus is needed on dynamic client factors, which may be targeted by interventions. Indeed, a recently developed framework of engagement in digital interventions incorporate both static and dynamic context factors as influencers of user engagement and acknowledges that engagement can be operationalized as including both usage (e.g., frequency of use, duration, etc.) and a user's subjective experience (e.g., attention, interest, etc.) (Perski et al., 2017). The operationalization of engagement will be dependent on the specific intervention and client characteristics (Perski et al., 2017). However, in determining the dynamic factors that may be relevant to understanding engagement, the field is severely lacking in theory‐driven approaches.

Glanz and Bishop (2010) highlighted that interventions based on empirical models are more effective than those which do not use a theoretical model, through offering a conceptual framework to explain causal processes. Further, Riley et al. (2011) argued that theoretically driven frameworks fit well with the rich data that online platforms provide. Despite the potential of theories to provide a rich understanding of user engagement, few studies to date have applied theoretical models to understanding issues of user engagement within digital mental health (Bull & Ezeanochie, 2016; Riley et al., 2011). However, one theoretical approach that has demonstrated some initial success in this field, is in the application of the theory of planned behavior (TPB).

### 2.2 The TPB

**Figure 1:**
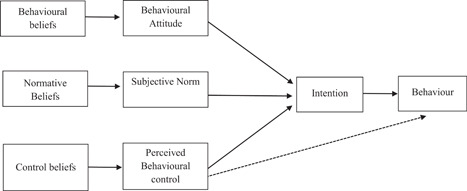
The theory of planned behavior (Ajzen, 1991)

The TPB is one of the most commonly cited models for predicting behavior in health settings (Clough et al., 2019). The theory highlights behavioral intention as fundamental to predicting action or enactment of a target behavior (Ajzen, 1991). Behavioral intention is formed based upon a person's attitudes toward the behavior, subjective norms, and perceived control over the behavior (Ajzen, 1987; Clough et al., 2019; Naslund et al., 2017). Behavioral attitude is a person's evaluation, either positive or negative, of the behavior (Ajzen, 1991). Subjective norms describe perceived social pressures to complete or not complete the behavior (Ajzen, 1991). Perceived behavioral control (PBC) refers to an individual's perception of volitional control over the behavior, that is, his/her concept of having adequate resources and opportunities to attempt the behavior (Ajzen, 1991). The role of PBC is multidimensional, having an independent influence on behavior but also interaction effects with subjective norms and attitude to influence behavior (see Figure 1) (Ajzen, 1991). The development of the three intention antecedents is a result of gathered information leading to beliefs about their function (Ajzen, 1991; Clough et al., 2019). An individual's salient beliefs are directly linked to the formation of attitudes, subjective norms, and perceptions of control (Ajzen, 1991).

The TPB model has been validated for the prediction of many health help‐seeking behaviors, including intention to seek face‐to‐face treatment for drug use (Booth et al., 2014) and depression (Bohon et al., 2016). However, as yet there has been limited research into its efficacy regarding digital mental health use.

To the authors' knowledge, only one study to date has applied the TPB model to understand digital mental health engagement (Clough et al., 2019). The study focussed on scale development (eTAP) using the TPB as a foundation. The eTAP was designed to help understand and predict ongoing engagement among clients following uptake of digital mental health interventions. The eTAP was developed as one scale in a suite of three, which focus on understanding and predicting client engagement in face‐to‐face therapy (TAP; Clough et al., 2017), digital therapies (eTAP; Clough et al., 2019), and therapist engagement with digital therapies (eTAP‐T; Clough et al., 2019).

The eTAP demonstrated strong psychometric properties, of which the overall model (four TPB factors) and intention (individual variable) were significant predictors of engagement with digital mental health interventions after 1 week (Clough et al., 2019). Engagement was measured dichotomously (yes/no) over a 1‐week period following completion of the measure. The scale predicted subsequent client engagement with 84% accuracy and non‐engagement with 74% accuracy. However, despite the initial support for the structural validity of the eTAP being consistent with the TPB, the factor structure of the measure was not validated in an independent sample. According to consensus‐based international guidelines for health instrument appraisal (Prinsen et al., 2018), structural validity that has only been validated in an exploratory manner can only be considered to provide “adequate” support for validity. “Very Good” supporting evidence for construct validity, argued as the second most important dimension of psychometric testing, can only be achieved through confirmatory analytical testing (Prinsen et al., 2018). Understanding and improving user engagement is only possible with the tools to accurately and sensitively measure the relevant constructs. While the eTAP has shown promise in this area, further validation of the proposed four‐factor structure is required. In addition, further investigation of the PBC factor of the eTAP is also needed.

Contrary to model predictions, PBC was not a significant individual predictor of user engagement in the original eTAP study (Clough et al., 2019). These results were surprising given that previous research in digital mental health and technology more broadly has highlighted the importance of users feeling comfortable and confident with the technological devices and programs (e.g., Hsieh et al., 2008). Given the TPB and PBC were operationalized in a pre‐digital world, their fit in digital environments may be moderated by factors unique to digital formats. One plausible factor which may not be captured by Clough et al.'s (2019) operationalization of PBC is eHealth Literacy.

Norman and Skinner (2006b) suggest that engagement with eHealth resources requires a distinct skill set or literacy of its own. eHealth literacy specifically describes a consumer's ability to search, find, understand, and appraise health information with the use of information technology, usually the Internet, to aid healthcare decisions (Eng, 2002; Norman & Skinner, 2006b). eHealth literacy may assist in capturing PBC in a digital context.

Within physical health research, higher eHealth literacy has been correlated with higher self‐efficacy in locating, understanding, and acting upon digital health information (Paige et al., 2017). Indeed, theoretically, both constructs are founded on control beliefs and are likely related. Yet, while both constructs relate to the capacity to perform a target behavior, eHealth literacy also includes additional components related to the capacity to understand and appraise online health information. The conceptualization of PBC in the eTAP mostly closely mirrors original behavioral conceptualizations of PBC and contains no items relating to a cognitive or critical understanding of online health materials. It is possible that the PBC scale in the eTAP was not a significant individual predictor of behavioral intention (Clough et al., 2019), as it may be a necessary but not sufficient condition to influence intention. That is, PBC may be moderated by eHealth literacy, with prediction being stronger when both are high; that the individual has both the skills to use online interventions (PBC) and the capacity to understand and apply this online health information (eHealth literacy). However, the possible role of eHealth literacy has not been explored within the area of digital mental health engagement, despite its potential to improve understanding of consumer engagement with digital mental health interventions.

### 2.3 The current research

Using tools to better predict and plan for consumer behavior is likely to have a large economic and social effect on digital mental health platform use. The eTAP is the only validated tool based upon the TPB in this area and importantly, forms part of a suite of tools that can be used to assess engagement in both face‐to‐face and digital interventions. Thus, the primary aim of this study was to assess the structural validity of the eTAP, by means of confirmatory factor analysis (CFA) in an independent sample. This step would further support the confidence in which the scale may be used to identify ways of improving client engagement. The second aim was to further investigate the role of PBC in predicting ongoing user engagement intentions, with eHealth Literacy considered as a possible extension to the original model. A number of predictions were made based on previous literature:

(1) It was hypothesized that the structural validity of the eTAP would be supported, with confirmation of the item model proposed by Clough et al. (2019). It was also predicted that the eTAP would demonstrate strong internal consistency for the total and subscales.
(2) Based upon the TPB, it was predicted that the three factors, behavioral attitude, subjective norm, and PBC, would each independently and significantly predict intention to continue engagement in digital mental health interventions, as would the overall model.
(3) It was predicted that eHealth literacy would moderate the relationship between PBC and participants' intention to continue engagement in digital health interventions. In particular, the relationship between PBC and intention would be strongest for participants with higher eHealth literacy compared to participants with lower eHealth literacy.

## 3 METHOD

### 3.1 Participants

The minimum sample size was determined based on field guidelines for linear modeling. The participant‐to‐variable ratio of 7:1 was used, as recommended by the consensus‐based standards for the selection of health measurement instruments (COSMIN; Prinsen et al., 2018). Hence, a recommended minimal sample size of 112 (7 × 16 eTAP items) was required.

Participants were adults aged 18 years and older, who resided in Australia, and were using a digital mental health tool at the time of recruitment. Restrictions were not placed on the type of digital mental health tool used, and recruitment materials identified that this could be an online program, app, website, forum, chat, videoconferencing, or virtual reality program. Restrictions were also not placed on frequency or duration of use, only that the participant identified as being a current digital mental health user. Initially, 498 participants were involved in a larger study investigating user perceptions of digital mental health, with 258 completing all items of the eTAP. However, 12 cases were subsequently excluded as the individuals reported they did not reside in Australia or were duplicate participant entries (*n* = 2). As such, the sample consisted of 244 participants, of which 182 were female (74.6%), 61 were male (25%), and 1 identified as other (0.4%). The age of participants ranged from 18 to 56 years (*M* = 22.80, SD = 7.67). Most (*n* = 154, 63.1%) reported their highest level of education as being high‐school and the sample was predominantly of Caucasian^1^/European ethnicity (*n* = 187, 76.6%). Of the sample, 232 provided details regarding the type of digital mental health intervention they were using. The majority of these (*n* = 160, 69.0%) were using mobile phone apps for mental health, with a minority using chat or telephone‐based services (*n* = 25, 10.8%), videoconferencing services (*n* = 7, 3.0%), online programs or websites (*n* = 6, 2.6%), or other digital mental health interventions (*n* = 34, 14.7%). Further sample characteristics are displayed in Table 1.

**Table 1:** Demographic frequency statistics

Variable	*n*	%
Highest level of education completed		
High school (up to and including Grade 12)	154	63.1
Trade/apprenticeship	1	0.4
Certificate/diploma	53	21.7
University degree	25	10.2
Higher university degree (e.g., Graduate Diploma, Masters, PhD)	11	4.5
Ethnicity		
Caucasian^a^/European	187	76.6
Asian	25	10.2
Middle Eastern	3	1.2
African	4	1.6
South Pacific Islander	1	0.4
Indigenous Australia	2	0.8
South American	9	3.7
Other	11	4.5
I prefer not to say	2	0.8

Abbreviations: *n*, numbers of participants, %, percentage of participants.

^a^ Term as presented in the online survey and as is common usage in the country of data collection.

### 3.2 Measures

#### 3.2.1 eTAP questionnaire

The eTAP (Clough et al., 2019), developed from the original TAP (Clough et al., 2017), was designed to measure factors related to ongoing client engagement (rather than initial uptake) in digital mental health interventions (also known as e‐interventions) for mental health. The eTAP consists of 16 self‐report items, designed to tap the four constructs of the TPB (intention, attitude, subjective norm, and PBC). Participants rate endorsement of each item (e.g., “I will use my online intervention for mental health in the next week”) using seven‐point rating scales ranging from 1 to 7 for intention, subjective norm, and PBC subscales, and −3 to +3 for attitude. Responses on the attitude subscale are rescaled (to a matching 1–7 rating scale) before scoring and interpretation.

The eTAP has demonstrated good structural validity through exploratory factor analysis, with the four emerging factors being consistent with those of the TPB (Clough et al., 2019). Reliability was also supported, with excellent internal consistency for the total scale (Cronbach's *α* = 0.92) and individual subscales (*α*s > 0.78). The eTAP demonstrated moderate 1‐week test–retest reliability (ICC = 0.72). The convergent (eTAP attitudes and Attitudes towards Psychological Online Interventions, *r* = 0.56, *p* < 0.01; eTAP subjective norms and Multidimensional Scale of Perceived Social Support, *r* = 0.33, *p* < 0.01; and eTAP PBC and Internet Self Efficacy, *r* = 0.29, *p* < 0.01), divergent (with likelihood of professional help‐seeking), and predictive validity (1‐week dichotomous user engagement) of the measure was also supported.

#### 3.2.2 eHealth literacy

eHealth literacy was measured using the eHealth Literacy Scale (eHEALS) (Norman & Skinner, 2006a). The eHEALS is an eight‐item scale in which participants rate their agreement to statements (e.g., “I feel confident in using information from the internet to make health decisions”) on five‐point rating scales, ranging from 1 “strongly agree” to 5 “strongly disagree.” The eHEALS demonstrated high internal consistency (*α* = 0.88) and test–retest reliability. Internal consistency in the current study was considered good (*α* = 0.90), and was comparable to results from the original study (Norman & Skinner, 2006a).

### 3.3 Procedure

Before commencing the research, ethical approval was obtained from the host university's human research ethics committee. Data were collected via an online survey. Participants were sampled through convenience and snowball sampling via online platforms, including mental health forums, social media, and the university's online research volunteer pool. Online informed consent was obtained before the commencement of the study. Participants from the general population were not provided with participation incentives, however, students from the university participant pool received partial course credit. The survey took approximately 10 min to complete.

## 4 RESULTS

### 4.1 Measuring the TPB

Data were analyzed using SPSS and Amos (Arbuckle, 2014; IBM Corp, 2019). CFA was conducted on the eTAP. Twelve cases were identified as being non‐engaged responders (e.g., all item responses were the same across the study) and were removed. Multiple eTAP items had negative skew and leptokurtic distributions. However, transformations are not recommended for CFA and as such these variables were not altered (Tabachnick & Fidell, 2013). Ten univariate and 10 multivariate outliers were identified. As CFA is sensitive to outliers, the analyses were run with (*n* = 232) and without (*n* = 212) these cases (Hu & Bentler, 1998). The exclusion of outliers had no significant impact on the interpretation of analyses, and as such, the outliers were retained (Tabachnick & Fidell, 2013). All other assumptions (e.g., linearity, multicollinearity) were met for each analysis, according to the guidelines of Tabachnick and Fidell (2013). Items and corresponding original subscales are displayed in Table 2 and descriptive statistics are displayed in Table 3.

**Table 2:** eTAP items and original corresponding subscales

Item number	Item	Subscale
1	I will use my online/digital mental health intervention for mental health in the next week	Intention
2	I find online/digital mental health interventions for mental health to be: (not helpful/helpful)	Attitude
3	Those people who are important to me would approve of me using online/digital mental health interventions for mental health	Subjective norm
4	I possess the required technical knowledge to use online/digital mental health interventions for mental health	Perceived behavioral control
5	It is likely that I will use my online/digital mental health intervention for mental health in the next week	Intention
6	Most people who are important to me would approve of me using online/digital mental health interventions for mental health	Subjective norm
7	I find using online/digital mental health interventions for mental health to be: (harmful/beneficial)	Attitude
8	It is mostly up to me whether I use my online/digital mental health intervention for mental health in the next week	Perceived behavioral control
9	I intend to use my online/digital mental health intervention for mental health in the next week	Intention
10	I find using online/digital mental health interventions for mental health to be: (unpleasant/pleasant)	Attitude
11	Those people who are important to me would support me using online/digital mental health interventions for mental health	Subjective norm
12	I intend to ensure I have access to the required technology to use my online/digital mental health intervention for mental health in the next week	Intention
13	I have complete control over whether I use online/digital mental health interventions for mental health	Perceived behavioral control
14	I find online/digital mental health interventions for mental health to be: (not credible/credible)	Attitude
15	I am confident using the technology for my online/digital mental health intervention for mental health	Perceived behavioral control
16	Those people who are important to me think online/digital mental health interventions for mental health are credible	Subjective norm

Abbreviation: eTAP, e‐Therapy Attitudes and Process.

**Table 3:** Correlations between eTAP items

Items	1	2	3	4	5	6	7	8	9	10	11	12	13	14	15	16
1.	‐	0.44**	0.21**	0.37**	0.82**	0.22**	0.37**	0.26**	0.80**	0.38**	0.27**	0.56**	0.28**	0.31**	0.36**	0.14*
2.		‐	0.39**	0.38**	0.46**	0.39**	0.67**	0.37**	0.50**	0.69**	0.46**	0.39**	0.40**	0.62**	0.46**	0.40**
3.			‐	0.42**	0.31**	0.82**	0.45**	0.37**	0.32**	0.44**	0.82**	0.38**	0.36**	0.42**	0.38**	0.64**
4.				‐	0.43**	0.39**	0.41**	0.48**	0.36**	0.41**	0.43**	0.47**	0.54**	0.44**	0.71**	0.34**
5.					‐	0.34**	0.47**	0.29**	0.84**	0.43**	0.35**	0.60**	0.31**	0.41**	0.42**	0.26**
6.						‐	0.53**	0.40**	0.36**	0.48**	0.88**	0.47**	0.42**	0.46**	0.41**	0.65**
7.							‐	0.49**	0.48**	0.66**	0.49**	0.48**	0.47**	0.70**	0.54**	0.43**
8.								‐	0.31**	0.37**	0.41**	0.39**	0.72**	0.44**	0.50**	0.35**
9.									‐	0.49**	0.43**	0.66**	0.35**	0.45**	0.46**	0.32**
10.										‐	0.56**	0.48**	0.47**	0.73**	0.57**	0.54**
11.											‐	0.47**	0.45**	0.54**	0.47**	0.70**
12.												‐	0.52**	0.41**	0.51**	0.32**
13.													‐	0.47**	0.62**	0.35**
14.														‐	0.57**	0.59**
15.															‐	0.43**
16.																‐
Mean	4.83	5.46	5.68	6.00	5.08	5.77	5.69	6.18	5.08	5.48	5.75	5.66	6.20	5.49	5.87	5.35
SD	1.61	1.15	1.46	1.28	1.60	1.45	1.07	1.24	1.59	1.24	1.41	1.35	1.24	1.14	1.31	1.35

Abbreviations: eTAP, e‐Therapy Attitudes and Process; SD, standard deviation.

* *p* < 0.05

** *p* < 0.01.

**Figure 2:**
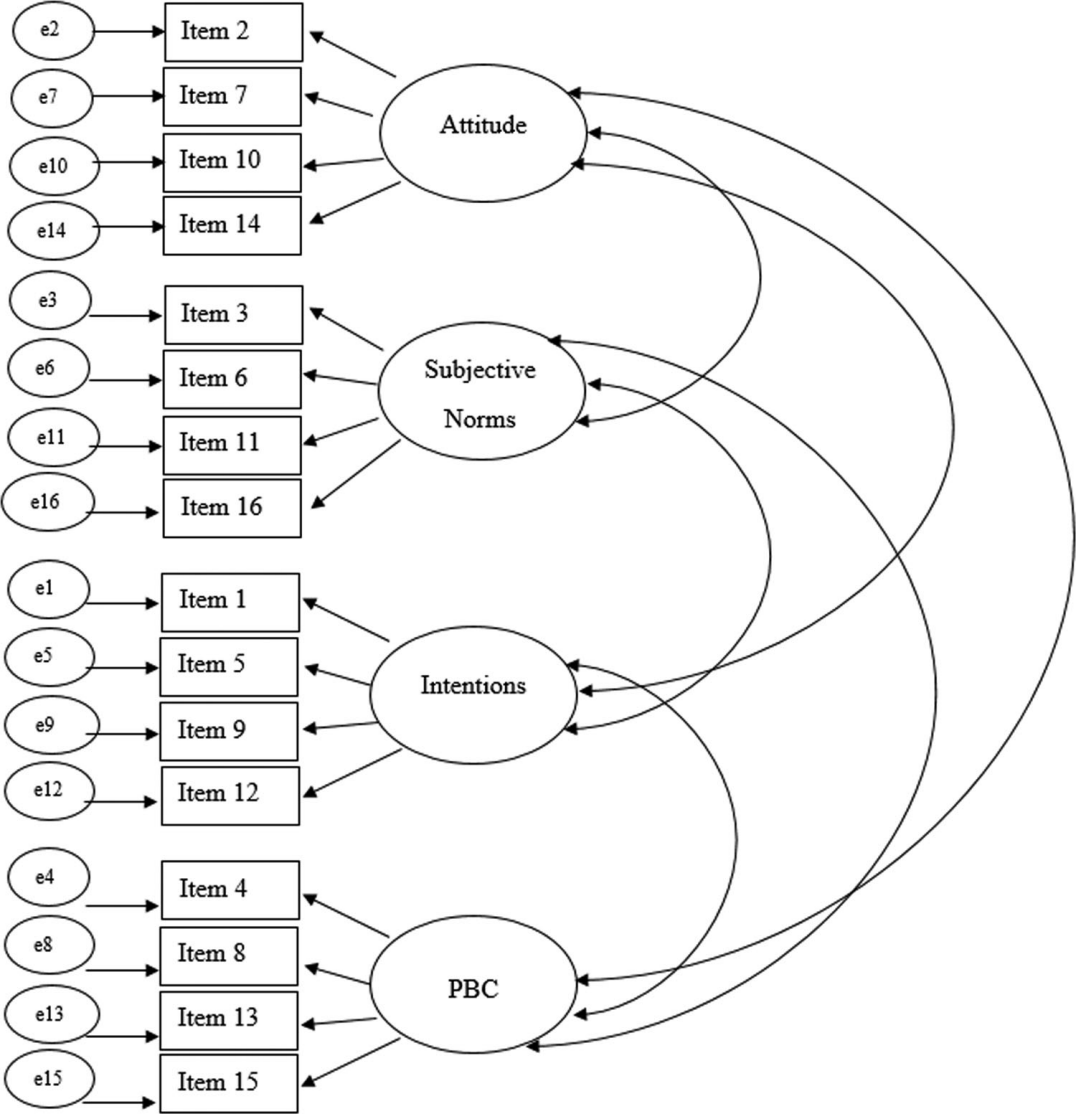
The proposed four‐factor confirmatory factor analysis pathway model

Model fit was determined based on examination of the *χ* ^2^ statistic and four relative fit indices: the Root Mean Square Error of Approximations (RMSEA); the Comparative Fit Index (CFI); the Normed Fit Index (NFI), and Standardised Root Mean Square Residual (SRMR). A four‐factor CFA was conducted (*n* = 232), as per the model displayed in Figure 2. The CFA did not produce a good fit (RMSEA = 0.10, CFI = 0.92, NFI = 0.90 SRMR = 007, *χ* ^2^ (98) = 341.16, *p* < 0.001). Following examination of the modification indices for the regression weights, the parameter between PBC and Item 12 was identified to have cross‐loading (Byrne, 2010). As Item 12 was postulated to measure intention, theoretically it was appropriate for this item to have a pathway with PBC as the intention is formed based on the three factors including PBC. Hence, the decision was made to allow for a direct path from PBC to Item 12. This modification improved the overall fit of the model (RMSEA = 0.10, CFI = 0.93, NFI = 0.90 SRMR = 0.05, *χ* ^2^ (97) = 304.55, *p* < 0.001).

**Figure 3:**
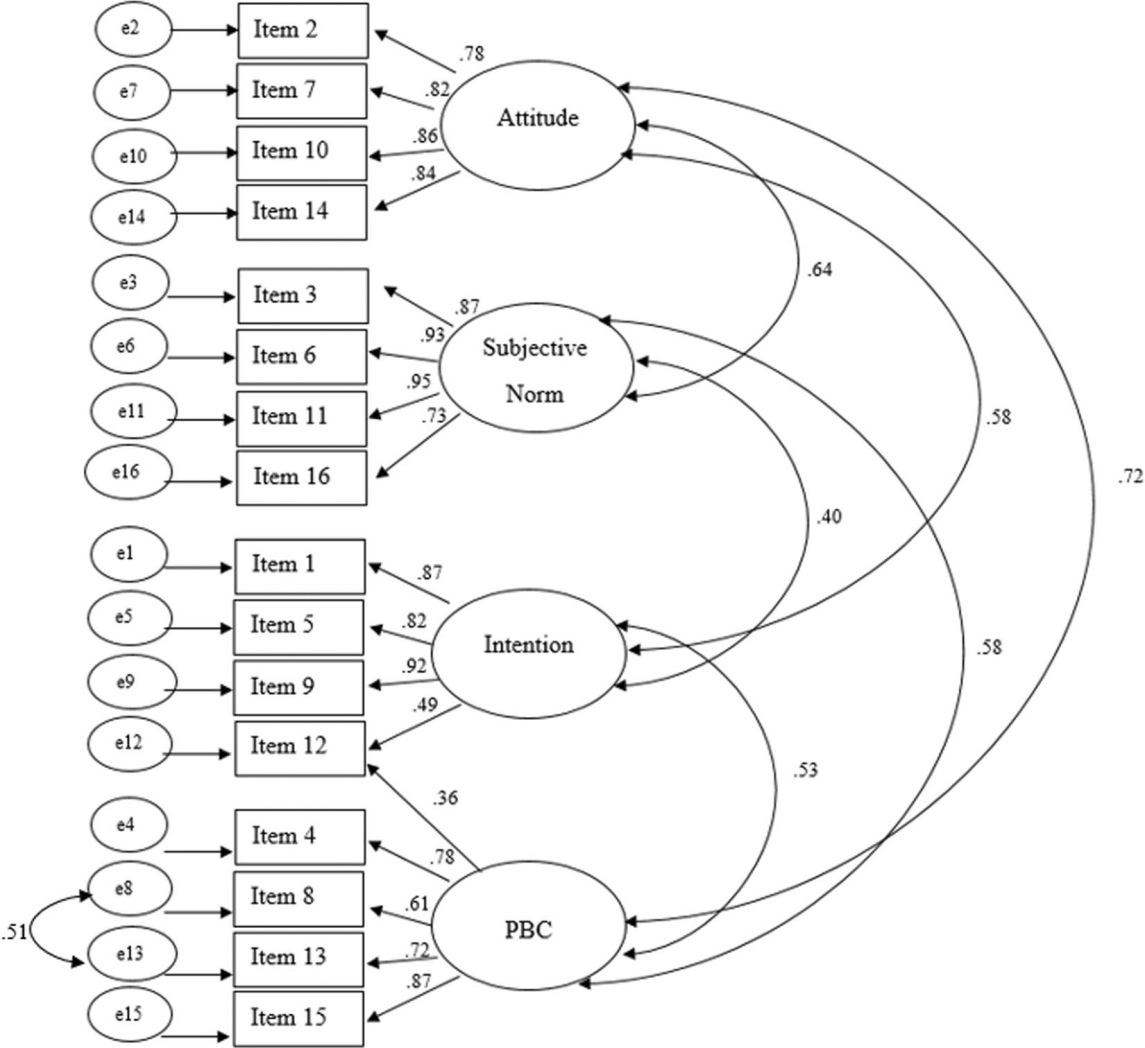
The final four‐factor final model with pathway modifications and regression weights

The modification indices were examined again as recommended by Byrne (2010) through a step‐by‐step process. Items 8 and 13 (on the PBC factor) were identified to have poor covariance. As this pair of items measured the same theoretical construct (PBC), the decision was made to allow for covariance between the items. This further modification improved the overall fit of the model. The final model (Figure 3) was a good fit across all four indices (RMSEA = 0.08, CFI = 0.95, NFI = 0.92, SRMR = 0.05, *χ* ^2^ (96) = 252.33, *p* < 0.001).

Internal consistency analyses for the total scale and individual subscales of the eTAP were conducted. Cronbach's *α* reliability coefficients were excellent for the total scale (0.93), intention subscale (0.91), and subjective norm subscale (0.92), and good for the PBC subscale (0.85) attitudes subscale (0.89).

### 4.2 Predicting intentions to use digital mental health

A total of 241 participants provided data on both the eTAP and eHEALS, which were entered into a hierarchical multiple regression. Deviations from normality were observed in the data. All analyses were run on untransformed, transformed, and transformed data with outliers removed. No differences were found, with the exception of age, which became a nonsignificant predictor when outliers were removed and transformed data were used. As this variable was not core to the research question, and all other analyses remained unchanged, untransformed data has been reported for ease of interpretation. All other assumptions were met. Descriptive statistics are displayed in Table 4.

**Table 4:** Descriptive statistics and correlations among constructs

Latent variable	1	2	3	4	5	6	7	8	9
Mean (standard deviation)	22.85 (7.69)	2.23 (2.29)	2.29 (1.29)	1.27 (.45)	22.20 (4.22)	22.57 (5.26)	24.16 (4.48)	20.81 (5.62)	31.12 (5.15)
1. Age		−0.58	0.589**	−0.86	0.206**	0.141*	0.145*	0.191**	0.127*
2. Ethnicity			−0.049	−0.068	−0.015	−0.094	−0.061	−0.125	−0.092
3. Education				−0.103	0.078	0.054	0.096	0.128*	0.113
4. Gender					−0.214**	−0.134*	−0.252**	−0.164*	−0.079
5 Attitude (eTAP)						0.644**	0.686**	0.604**	0.368**
6. Subjective norms (eTAP)							0.582**	0.452**	0.253**
7. PBC (eTAP)								0.555**	0.387**
8. Intention (eTAP)									0.322**
9. eHealth literacy									

Abbreviation: eTAP, e‐Therapy Attitudes and Process; PBC, perceived behavioral control.

* *p* < 0.05

** *p* < 0.01.

Hypotheses were explored in a three‐step method of hierarchical multiple regression to investigate predictors of intention. Step 1 entered the demographic variables of interest which had been found to significantly correlate with intention (age, gender, and education). In Step 2 the predictor variables of the original TPB model (attitudes, social norms, perceived behavioral) were entered, as was eHealth literacy. The capacity of these variables to predict intention, beyond that of the previously entered demographic variables, was examined. The interaction term (PBC × eHealth literacy) was entered in Step 3.

At Step 1 of the analysis demographic factors accounted for a significant proportion of variance (6%) in intention to continue using digital mental health tools. At this stage of analysis, age and gender each accounted for approximately 2% of the unique variance in intention. Older age and being female were predictive of greater intention to use digital mental health tools. Education was not a significant individual predictor of intention. See Table 5, Model 1 for a summary of results.

**Table 5:** Summary of multiple regression and moderation analysis for variables predicting intention to continue using digital mental health interventions

	Model 1						Model 2					Model 3				
	Variable	*B*	Se*B*	*Sr* ^2^	*t*	*p*	*B*	Se*B*	*Sr* ^2^	*t*	*p*	*B*	Se*B*	*Sr* ^2^	t	*p*
Demographic variables	Age	0.172	0.057	0.019	2.202	0.029	0.039	0.046	<0.001	0.614	0.540	0.040	0.046	0.001	0.632	0.528
		(0.013, 0.238)					(−0.063, 0.120)					(−0.062, 0.121)				
	Gender	−0.148	0.789	−0.022	3.56	0.020	−0.006	0.650	<0.001	−0.112	0.911	−0.008	0.650	0.001	−0.147	0.883
		(−0.618, −0.720)					(−1.352, 1.207)					(−1.377, 1.186)				
	Education	0.012	0.340	<0.001	0.151	0.880	0.042	0.272	0.001	0.677	0.499	0.049	0.274	0.002	0.779	0.437
		(−0.618, 0.720)					(−0.352, 0.720)					(−0.327, 0.754)				
Test variables	Attitude						0.375	0.103	0.059	4.83	<0.001	0.369	0.104	0.056	4.74	<0.001
							(0.296, 0.702)					(0.287, 0.696)				
	Social norms						0.050	0.073	0.001	0.736	0.463	0.045	0.073	0.001	0.659	0.463
							(−0.090, 0.196)					(−0.096, 0.192)				
	PBC						−0.230	0.093	0.024	3.117	0.002	0.444	0.321	0.007	1.74	0.084
							(0.106, 0.471)					(−0.075, 1.191)				
	eHealth literacy						0.073	0.060	0.004	1.319	0.189	0.283	0.269	0.003	1.148	0.252
							(−0.039, 0.198)					(−0.221, 0.840)				
Interaction	PBC × eHealth literacy											−0.352	0.011	0.002	−0.875	0.383
												(−0.031, 0.012)				
Model statistics	*R* ^2^	0.059					0.414					0.416				
	*R* ^2^ change						0.355					0.002				
	*F*	4.92				<0.01	23.48				<0.001	20.62				<0.01
	*F* change						35.27				<0.001	0.766				0.383

Abbreviation: PBC, perceived behavioral control.

The combined variables in Model 2 accounted for significantly greater variance than that previously accounted for in Model 1 (Table 5, Model 2). The overall model explained approximately 41% of the variance in intention to use digital mental health technologies. Both attitude and PBC significantly predicted intention. Positive attitude toward the technology predicted stronger intentions to use digital mental health and was the most important individual predictor within the model, accounting for 6% of unique variance. PBC was also a significant predictor of intention, with greater PBC predicting stronger intentions to use digital mental health, accounting for 2% of unique variance in intention. Social norms and eHealth literacy were not significant individual predictors of intention.

At Step 3 of the analysis, the addition of the interaction term did not significantly improve prediction of intention from Model 2, nor was the interaction term a significant individual predictor, accounting for an increase of less than 1% in the model's ability to predict intention (Table 4, Model 3). Examination of individual predictors within the moderation model showed that only attitude remained a significant predictor of intention, with its explanation of unique variance remaining stable (6%). Within this model, neither demographic predictors, remaining TPB factors (social norms, PBC), or eHealth literacy were significant individual predictors of intention.

## 5 DISCUSSION

The purpose of the present study was to investigate factors influencing client engagement intentions in digital mental health interventions, utilizing the framework of the TBP. The study aimed to provide confirmation of the structural validity of the eTAP in an independent sample. A secondary aim was to further investigate the role of PBC and eHealth literacy in predicting intentions to use digital mental health. Hypotheses regarding validation of the eTAP were supported, providing further confidence in the use of the scale. Results regarding PBC and eHealth literacy were mixed.

### 5.1 Scale validation

Based on the theoretical underpinnings of the eTAP it was hypothesized that the four factors would map onto the constructs of the TPB: intention; attitude; subjective norm; and PBC. A four‐factor model was obtained, which provided a good fit across multiple indices following two modifications. Both modifications were considered to be theoretically sound. Modifications are considered appropriate within CFA analyses when they are plausible, justified, and based on theoretical reasoning, as was the case in the current analyses (Byrne, 2010). Based on these results, and that the TPB argues covariance between constructs, it is maintained that modifications to scale scoring or interpretation of the original eTAP are not needed or appropriate at this time. However, users of the scale should consider the potential shared variance between factors, noting that a person's score on one dimension likely influences their scoring on other TPB dimensions. The eTAP also demonstrated excellent reliability for the full scale, as well as good to excellent reliability for the individual subscales. Further, the internal consistency of the full scale and subscales was comparable to coefficients reported by Clough et al. (2019). As such, both the structural validity and internal reliability of the measure were considered to be supported.

Given the lack of validated and theoretically based scales to understand and predict client engagement with digital mental health interventions, this independent evaluation provides greater confidence in the use of the eTAP within this field. Indeed, given the tool's previous success in predicting client engagement, it is anticipated that it will prove useful for accurate measurement, identification of at‐risk clients, and development and evaluation of interventions to improve client engagement in digital mental health programs. The tool's place within a suite of measures will also enable comparison of engagement factors across face‐to‐face (TAP), digital (eTAP), and therapists' engagement with digital interventions (eTAP‐T) in future research. Such research may enable the development of interventions to support client engagement, that are tailored to the modality of intervention delivery. Such a study would provide further insight into the mechanisms important for fostering engagement with digital mental health tools at the clinician and client level, as well as further insight into possible process differences between face‐to‐face and digital interventions.

### 5.2 Predictors of intention

Overall, there was partial support for the efficacy of the TPB model and no support for the addition of eHealth literacy as a moderating predictor of PBC. Attitude was a strong and independent predictor of intention to continue using digital mental health tools, while social norms and demographic predictors (age, gender, and level of education) were not consistent predictors of intention. These results reinforce the need for theoretically driven approaches to understand issues of client engagement. Indeed, client attitudes will likely be of particular importance for interventions to improve engagement with digital mental health interventions. The results were consistent with the previous study by Clough et al. (2019), which also found attitudes and intentions to be the most important model predictors of subsequent behavioral engagement.

Digital mental health approaches are typically perceived by clients as being less efficacious than face‐to‐face approaches (Norwood et al., 2018), and this attitude has been amplified amidst the COVID‐19 pandemic (Waller et al., 2020). In attempting to meet the increased demand for mental health services as a result of the pandemic (Javed et al., 2020), interventions to improve attitudes towards digital mental health will be more important than ever before (Taylor et al., 2020). These results provide direction regarding intervention development. Specifically, interventions to improve ongoing client engagement should focus on targeting client beliefs and attitudes regarding digital mental health programs. Approaches aimed at improving clients' commitment to behavioral enactments, such as motivational interviewing approaches, will likely also be beneficial to improve clients' behavioral intentions to engage in digital mental health.

PBC and eHealth literacy were not reliable predictors of intention for ongoing digital mental health engagement in this study. These findings were inconsistent with previous research citing the importance of comfort and confidence with technology for engagement (March et al., 2018). It is possible that these factors may be less important predictors for ongoing engagement versus the initial uptake of digital mental health technologies. That is, participants were already engaged in digital mental health tools and hence may have already overcome barriers to access. Data attained from contemplative stage individuals may provide different results as to the influence of PBC and eHealth literacy on the intention for initial use of digital mental health tools. Another possible explanation concerns the eHEALS' focus on broad health literacy, which may not be nuanced enough to capture the digital and health literacy skills important for engagement in digital mental health interventions. Mental health literacy has proven an important construct in predicting engagement with psychological interventions more broadly (O'Connor et al., 2014). It is possible that a similar “e‐mental health literacy,” combining digital and mental health literacy constructs, may be more appropriate for understanding engagement with digital mental health tools.

Social norms also did not have a significant individual influence on intention to continue digital mental health use, which was consistent with previous research identifying social norms as having less predictive power than other variables of the TPB model (Karahanna et al., 1999; Russo et al., 2015). Within the context of digital mental health, conforming to social norms may be less pertinent due to the high level of discretion possible in accessing these services. For ongoing use, the current study highlights that the perceptions arising from experience using the technologies (attitudes) are of most importance for predicting ongoing user engagement with digital mental health interventions.

### 5.3 Limitations and directions for further research

A number of limitations should be considered within the current study. First, the data set was skewed toward females and persons from Caucasian and higher education backgrounds. Although results may not be representative of the general population, females are often found to be a higher help‐seeking population, and as such this skew may still be representative of typical help‐seeking populations (Nam et al., 2010). Given the focus on better understanding ongoing engagement with digital mental health interventions, it should be noted that the present findings may also not be generalizable to understanding uptake or initial engagement. Future studies may benefit from considering the theoretical factors of importance at different stages of help seeking.

Results should also be considered in the context of the study not including a measure of actual behavioral engagement, only participants' intentions to engage. As such, the extent to which behavioral intentions (as measured by the eTAP) accurately predict ongoing engagement with digital mental health interventions was not able to be investigated in the current study. Although predictive validity was reported in the original validation study (Clough et al., 2019) and has considerable support in the broader TPB health prediction field (e.g., McEachan et al., 2011), future research should aim to provide further support for this in independent and diverse samples. Similarly, the current research replicated the original study by utilizing a sample of current users of digital mental health. Future research should focus on establishing the validity of the scale among diverse populations, including users who have “dropped out” of digital mental health interventions. This avenue may prove useful in understanding the theory‐based factors influencing client decisions to remain or leave digital mental health interventions. Finally, this study utilized convenience sampling. Participants with higher pre‐existing digital and e‐health literacy and positive attitudes towards digital interventions may be more likely to have responded to the online questionnaire. This self‐selection may therefore have impacted the results, particularly regarding attitudes towards digital interventions and perceptions of barriers (PBC). Future studies may benefit from using more purposive sampling methods.

## 6 CONCLUSIONS

Digital mental health interventions are now widely available across disorders and therapeutic approaches, with considerable evidence available to support their efficacy (e.g., Andersson & Cuijpers, 2009; Firth, Torous, Nicholas, Carney, Pratap, et al., 2017; Firth, Torous, Nicholas, Carney, Rosenbaum, et al., 2017). Yet greater attention is needed in fostering ongoing client engagement with these interventions. The current study has provided further validation for one of the few theoretically based and psychometrically sound tools for understanding patient engagement intentions in digital mental health. When used in conjunction with validated frameworks of engagement in digital interventions (e.g., Perski et al., 2017), it may provide direction for the development of interventions to improve client engagement. The TPB model significantly predicted client intentions to continue using digital mental health intervention. Future research should focus on linking the TPB model and intentions specifically to digital mental health use. The eTAP will likely prove to be useful in both clinical and research contexts within digital mental health, particularly for comparisons across treatment modalities.

The importance of attitudes in predicting intentions is of particular relevance for clinicians and researchers. Clinicians who refer clients to digital mental health interventions should take care to address clients' attitudes and intentions for program use and provide support, psychoeducation, and intervention as needed. Furthermore, digital mental health interventions should not only focus on improving mental health and wellbeing but critically, should address client perceptions and attitudes toward the intervention. Pre‐interventions to improve client engagement in face‐to‐face mental health interventions have shown benefits (Ogrodniczuk et al., 2005), and although not yet tested, may be an important avenue for future research in digital mental health. Brief educational interventions have shown efficacy in shaping client attitudes toward digital mental health (e.g., Casey et al., 2013) and warrant further investigation for improving ongoing engagement in programs. Further research is also needed to clarify the importance of model factors across stages of help‐seeking and engagement. Indeed, as individuals often self‐select into digital mental health programs, the capacity of the TPB for improving attitudes at a population level to increase uptake of these interventions should also be a focus of future research. In the context of a predicted global mental health crisis (Zhou et al., 2020), interventions to improve the use of digital mental health interventions will be of particular importance in coming years.

Client engagement is a critical factor affecting the implementation and clinical success of digital mental health interventions, with dose–response patterns found between adherence to the program and treatment outcomes (Ghaderi, 2006; Karyotaki et al., 2017; Manwaring et al., 2008). Hence, the importance of exploring avenues to improve engagement in interventions is critical to ease the strain on national healthcare programs and improve population mental health. It is hoped that the current findings provide a starting point to understanding these process factors and will promote research and intervention development in this area.

## CONFLICTS OF INTEREST

The authors declare no conflicts of interest.

## AUTHOR CONTRIBUTIONS

Bonnie Clough contributed to study design, data analysis, and communication; Christina Yousif and Sophia Stillerova contributed to study design, data collection, data analysis, and communication; Sasha Miles and Aarthi Ganapathy contributed to study design, data analysis, and communication; Leanne Casey contributed to study design and communication.

### PEER REVIEW

The peer review history for this article is available at https://publons.com/publon/10.1002/jclp.23342

## ETHICS STATEMENT

This study was approved by the Griffith University Human Research Ethics Committee with participants' written informed consent received and archived.

## Data Availability

The data that support the findings of this study are available on request from the corresponding author. The data are not publicly available due to privacy or ethical restrictions.

## ENDNOTE

1 Term as presented in the online survey and as is common usage in the country of data collection.
